# Reviewing challenges and gaps in European and global dementia policy

**DOI:** 10.1108/JPMH-02-2018-0012

**Published:** 2018-12-17

**Authors:** Toni Wright, Stephen O’Connor

**Affiliations:** England Centre for Practice Development, Canterbury Christ Church University, Canterbury, UK

**Keywords:** Dementia policy, International perspectives, Marginalized peoples, Public health priorities

## Abstract

**Purpose:**

The purpose of this paper is to scope out European and global policy documents focused on dementia with the purpose of providing a synthesis of the challenges the phenomenon poses and the gaps evident.

**Design/methodology/approach:**

An adapted PESTEL framework as a data extraction tool resulted in an analysis of the political, economic, social, technological, environmental, organisational, educational and research aspects of dementia policy.

**Findings:**

Policy documents showed variability of dementia strategy, plan and programme development. All documents recognised rapidly growing ageing populations, and increasing numbers of people living with dementia. Dementia as a public health priority is inconsistent in growth. Global policy documents stress the impact of dementia will be felt most by low- and middle-income countries. Main themes were: a need to raise awareness of dementia and action to reduce stigma around it, the need for early diagnosis and preventative person-centred approaches with integrated care, fiscal investment, further research, training and education for workforces, increased involvement of and support for people living with dementia and care and support close to home.

**Practical implications:**

By identifying current dementia challenges and policy gap implications this analysis urges engagement with broader frames of reference as potential for enabling bolder and radically better dementia care models.

**Originality/value:**

This paper offers a review of present global and European dementia policy, outlining the potential implications for the most marginalised in society if it fails to be critical of its own underpinning assumptions.

## Background

### Aim and purpose

The aim of this review is to scope out European and global policy documents focused on dementia with the purpose of identifying and providing a synthesis of the challenges which the phenomenon poses across populations and regions, as well as the gaps evident in existing policy.

### Search strategy

A desktop search concentrated on finding international and national documents focused on European and global dementia policy from 2010 onwards. Where various versions of policy documents were uncovered, to maintain timeliness, the superseded versions of documents were not considered. The search terms used are listed in Box 1. A total of 37 relevant policy documents were discovered. In total, 28 related to European country contexts and 9 to world/global overviews. Of the 28 European policy documents, 13 had no full English version[1], although there were brief English summaries available via web content, so these are included in the review. The remaining 15 European documents had full English versions[2]. All of the nine world/global documents had full English versions.

Box 1 **Search terms**

European dementia policy

Global dementia policy

Policy +

+ Neurological disease

+ Dementia

+ Alzheimer

## Data analytic procedures

This policy review was undertaken using an adapted PESTEL framework (Aguilar, 1967; Johnson *et al.*, 2005) as a data extraction tool. The original PESTEL analyses political, economic, social, technological, environmental and legal aspects of a phenomenon. For this review, legal phenomena have not be adopted, but organisational, educational and research considerations have been added resulting in a PESTOEER (political, economic, social, technological, organisational, environmental, educational and research) framework. The exclusion and additions were necessary because they reflect the content that emerged from the policy documents. An organisational understanding enables consideration of impacts and implications for structural and administrative health and social care services, models and approaches. Educational and research dimensions are equally essential to include as they offer insight into issues around education and training for workforce development and details of the current research context. The adapted framework acts as an analytical tool that enables categorical content analysis (Boos and Tarnai, 1999) of the policy documents.

Three principles where employed when reviewing the discovered policy documents:

1. To identify categorical content data that fitted with or added to the PESTEL framework. This resulted in the emergence of the PESTOEER framework, from where common themes could be identified across the policy documents.
2. Assessment of the sophistication and development of the policy document, so the extent to which it was in preliminary or draft stages or had been fully implemented and evaluated. This enabled a mapping out of dementia strategy, plan and programme development.
3. Assessment of evidence that the policy documents reflected a social justice perspective, in the sense that the most socially marginalised and vulnerable (women, LGBTQIA+, disabled and Black and minority ethnic people) living with dementia had their needs mentioned, explored or advocated for.

The following sections that summarise and provide a gap analysis based on the categorical content analysis of the discovered policy documents are reflective of the three principles underpinning the review process.

## Summary of policy documents

The European documents reviewed varied greatly, from a paragraph description to full length detailed papers of national strategies and plans. This variation is reflective of the difference across European countries in degree of development in dementia strategies, plans and programmes; and the degree to which they are implemented, monitored and measured. At the time of writing, very few European countries amongst the members of Alzheimer Europe[3] had no national strategy policy documents relating to dementia, although the landscape of development is rapidly changing and all were either in development, lobbied for, or had political backing[4]. There are significant differences between with European and global policy documents, with the European ones honed specifically for individual countries and the global perspective more attentive to the disparities and inequities between the Global North and Global South.

All documents stressed the importance of dementia needing to be seen as a public health priority. The main themes emerging from the documents are presented in Tables I–IV.

All documents recognised the rapidly growing ageing populations globally, and as a consequence, the increasing numbers of people living with dementia (including families and carers), with the global perspective documents clearly articulating that this will have the most significant impact in low- and middle-income countries (World Health Organisation, 2012, 2017; Prince *et al.*, 2013; Age International, 2014; Rubinstein *et al.*, 2015; Corfield, 2017).

Across the European policies there is great variability in terms of dementia being a public health priority, but this is unsurprising given that historically the link between dementia, public health and policy has been slow to develop (Williamson, 2015). This variation spans from draft (Age International, 2014) to full and final strategies and plans (Dementia Services Development Centre The University of Stirling, 2011; Brodsky *et al.*, 2013; Ministry of Social Affairs and Health Finland, 2013; Age International, 2014; Alzheimer Europe, 2014, 2015, 2016a, b, c, d, e, f, g, h; Di Fiandra, 2014; HM Government of Gibraltar, 2015; Scerri, 2015), including those beginning to be implemented (Ministry of Health, Welfare and Sport *et al.*, 2009; Alzheimer’s Society, 2011; Alzheimer Europe, 2016i; Norwegian Ministry of Health and Care Services, 2016; Hanselmann *et al.*, 2014), and those having been implemented and now being monitored (Scottish Government, 2013) (Scotland). Bosnia and Herzegovina (Alzheimer Europe, 2017), Germany (although the states of Bavaria and Saarland do have strategies (Alzheimer Europe, 2016j), Romania (Alzheimer Europe, 2017), and Turkey (Alzheimer Europe, 2017) have no national strategy or plan, although, as mentioned before, having no strategy does not always equate to no pressure being brought to bear for one to be developed. In Germany and Turkey, in particular, advocacy groups are lobbying their governments around dementia strategy development. Despite each of the published documents predicting a potential looming dementia crisis, it is generally accepted that dementia is not yet seen by many governments as a public health priority. The World Health Organisation (2017) are clear that for some governments it is simply not on their agenda at all.

The global policy documents focus on global regions rather than nation states. They point out that the fastest developing populations living with dementia are predicted to be in the low- and middle-income countries that tend to be located in the Global South. Also Keenly stressed is the likelihood of growing health inequities between populations living in the Global North compared to those in the Global South (WHO, 2012, 2017; Prince *et al.*, 2013; Age International, 2014; Rubinstein *et al.*, 2015; Corfield, 2017). There is a call for action to be taken to lessen inequities between global regions through reducing the cost of medications in the Global South in exchange for conducting medical trial sites and investment in health infrastructure (The Alzheimer Society of Ireland, 2012; World Dementia Council, 2014). Within the global policy documents there is a lack of articulation around what individual country needs may be, and in that sense, a strong tendency to homogenise regions. The global policy documents do recognise the need for gender sensitive approaches to dementia strategies, plans, and programmes because women currently, and are likely to continue to, bear the largest burden as the main informal unpaid care providers. Also, women are most at risk of developing dementia themselves because they make up a larger proportion of older adults (Corfield, 2017; WHO, 2017). The documents are also heavily reflective of an orientation around western approaches and knowledge, meaning there is a paucity of knowledge and perspective coming directly from countries located in the Global South, giving a sense that those countries lack agency with regard to their futures.

**Figure 1:**
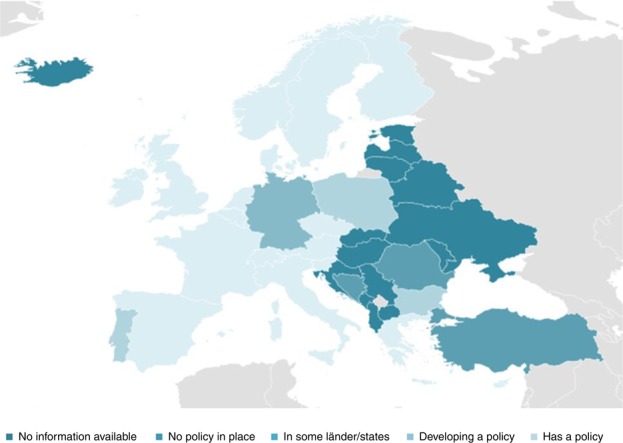
Countries with, without and currently developing dementia policies

Most of the policy documents discuss the future likely impacts of dementia, the associated challenges around these and commitment to seeing those challenges as a priority. Despite the rhetoric of predicted dementia crisis, concrete actions being taken to minimise the challenges of dementia are sparse and inconsistent both across Europe, as well as further afield as demonstrated in Figure 1.

## Policy gaps identified

The following main areas of concern have been identified as neglected or under-acknowledged within the discovered policy documents.

Generally, there is recognition across the policy documents of the main challenges dementia poses, and there are many suggestions as to what actions might be taken to limit the impact of those challenges. However, the predicted challenges and suggested antidotes tend towards narrow parochialism in the European policy documents that only focus on national impacts and solutions. The global policy documents are the antithesis to the European documents since they take a broad divided Global North/Global South perspective, in which they often fail to account for the vast number of political and economic differences in nations located within those large geographical areas or regions. There is therefore, a disconnect between the European and global documents in that they do not “talk” to each other and seem to be developed in isolation, so that there is no consideration of how local or national European agendas and schemas fit with or are informed by wider global challenges and vice versa. Consequently, whilst it is recognised that individual governments need to be responsible for policies to address the challenges dementia poses, there is little acknowledgement of the contribution that specific European or global strategies might make to the oversight and accountability mechanisms, processes and structures which enable policy to do the work it is intended to do, namely, address global challenges and continue to respond and develop proportionately and appropriately. Whilst suggestions for limiting the negative impact of dementia are proposed, it is unclear in many cases whether the rhetoric results in action, and where it does; to what degree it is implemented. Where full strategies, plans and programmes do exist, their implementation is for the most part only just beginning, and as a consequence their impact has not yet been fully evaluated.

Across all policy documents there is very little mention of the contribution that technology could make in easing the burden which dementia poses, such as assistive technologies to enhance quality of life of those living with dementia, or augment and improve the way in which health and social care workers deliver care and provide support. Some documents (Prince *et al.*, 2013; World Dementia Council, 2014; Rubinstein *et al.*, 2015; Alzheimer Europe, 2016e) acknowledge the prospective potential of using robotics to support health care delivery, supplement the dementia workforce and change care environments, but this is not a central theme for any policy documents, and in general, consideration of technological advances and their contribution to dementia care is under-explored within all of the policy documents.

Political, economic and environmental aspects are also sparsely attended to across the policy documents. Politically, most talk is about the importance of dementia being treated as a public health priority. Economically, the documents invariably express a need for substantial financial investment. However, the means by which investment could be raised or made available, and the way in which this would be distributed across various areas (country, continent or global regions) is not articulated. Calls for political action and monetary investment are premised on the assumption that the political will and financial means to invest in dementia care actually exist, whereas these are seldom evidenced. There is moreover, no direct reference to environmental factors within any of the documents, so implications of the policies environmental impact on pollution, land ethics, biodiversity and local/national ecologies are not considered. For example, the environmental impacts from building new specialist/expert care facilities, dementia housing^/^communities, pharmacological research and development, etc. are excluded from the equation, and no documents indicate the need for green ideology to play a role in tackling the challenges of dementia, or for attention to the effects of climate change and what that will mean for how and where people live. No new and/or radical interconnected political, economic, social or environmental models are proposed as possible futures.

European policy pays little attention to the needs of, and implications for, Black, Asian and minority ethnic people, LGBTQIA+ people or other marginalised groups, such as those living with learning and physical disability, those living with mental ill health and those, especially women, living in poverty. Some global policy documents do focus on increasing negative dementia implications for those living in the low- and middle-income countries, especially women in those countries, and therefore advocate the need for gender sensitive approaches to policy development, health care and social support. The failure of policy strategies, plans and programmes to explicitly make reference to the needs of the most disadvantaged and vulnerable across societies means their very particular needs remain marginal rather than central. By not paying attention to those very particular needs, policies, plans and programmes run the risk of not engaging critical social justice perspectives that could enable radical and far-reaching shifts in the approaches and understanding of the challenges of dementia (Hulko, 2011).

Much of what is advocated for in terms of safe and effective health and social care provision is reflective of current thinking across existing health and social care pathways about what enables good quality care and support. As such, the documents advocate for the well-rehearsed and accepted philosophies of integrated health care and social support systems, improved quality, preventative approaches, and specialist professionals. It is not always clear however (although some European documents do provide a good deal of detail), what such provision would actually looks like, how it would be enabled, and how it would be recognised and measured. There is a lack of acknowledgement throughout the documents that the implementation of safe, effective, person-centred care in dementia is a complex and challenging process (Clissett *et al.*, 2013), the delivery of which goes well beyond the simple implementation of policy or greater financial investment and requires wholesale systematic change to be effective.

There is a general consensus around taking a balanced way forward for dementia health and social care support that combines a social model approach with medical based solutions. Broadly, there is focus on improving quality of life for those living with dementia, but this runs in parallel with the pervasive pursuit of a cure and better medical treatment as answers for the challenges posed by the disease. This indicates that the medical model is still strongly present within policy positions going forward. However, missing from the policy documents is an understanding of how beholden a medical model approach is to large pharmaceutical companies who drive curative research and development for their own profits and political agendas. Some global documents stress the rights of low- and middle-income countries to affordable treatments and medications. In practice historically this has often meant pharmaceutical companies offering less expensive medications to low- and middle-income countries in exchange for drug trails. Such exchanges, especially when they involve women and poor populations (Crane and Dusenberry, 2004; Pugh *et al.*, 2017; Singh and Karim, 2017), have rightly been criticised as deeply unethical (Ellis, 2006; Kent, 2015).

Overall, across all the documents, there seems to be support for solutions to the challenges of dementia that fit within existing Eurocentric (Western) political, economic, social and philosophical frames of reference. In other words, there is an absence throughout of critique around the dangers of existing overarching structures, leaving us wanting of any original and radically innovative (Santos, 2014) models or systems of care.

## Policy gap implications

Variation in the rate of development of dementia strategies, plans and programmes will mean that different countries progress at different rates and hence tackle the challenges of dementia inconsistently. These variations provide potential for “early adopters” or emerging forerunners in the field to provide a valuable learning resource for those countries following in their wake, although solutions which work in one country may not necessarily work in another. They do, however, provide a useful benchmark and potential template against which the consideration of new strategies, plans and programmes can be predicated.

Those marginalised and vulnerable groups highlighted so well in the global strategies are unlikely to have their needs fully met at a national level unless there is commitment to affirmative action that listens to their voices, experiences and needs; and takes action in response to them (Hulko, 2009). Centring the experiences of marginalised groups allows policy agendas to expand for the benefit of all.

Inadequate consideration of the over-reliance on informal unpaid (and usually female) carers to provide care to people with dementia across a wide variety of countries, cultural contexts, and health and social care systems, means that some of these policies are likely to meet with varying degrees of success. The burden of dementia for those providing informal unpaid care is likely not only to remain considerable, but to grow exponentially over time. In some cases, this coincides with increasing pressure for women to join the job-market or engage in other economic forms of work, so the additional burden of care in most instances is going to be carried by women, and particularly women living in the Global South.

Without adequate health and social care infrastructures and without international efforts to share the wealth of high-income countries, the strategies, plans and programmes posited for low- and middle-income countries are unlikely to come to fruition. There are likely to be continuing large scale inequities between Global North and Global South populations’ experiences of care provision, with marginalised groups from the Global South affected the most by these inequities. Moreover, a lack of intercontinental vision, will, and commitment towards international collaborative and collective action oriented at meeting the needs of the most vulnerable and marginalised groups in the world is likely to cause greater dissonance between European and global policies and the outcomes from their implementation.

Europe has dominated much of the dementia policy discourse to date, and as forerunners in the field, along with the USA under the Obama administration (Hoang *et al.*, 2015), European governments may potentially (if they have not done so already) disregard many alternative, imaginative and innovative systems and models of care coming out of the Global South. A lack of vision around systems and models of care outside of mainstream political, economic, social and philosophical frames of reference will hinder the emergence of radically different approaches that could better meet the challenges and impacts of dementia (Hulko, 2011).

Continued lack of consideration of the impending environmental factors such as climate change – the effects of which are already being seen – will lead to population crisis points globally, with those living in the Global South feeling the effects of this more acutely (Sealey-Huggins, 2017). Responses to these factors are likely to be reactionary and crisis driven, leaving the most vulnerable and marginalised most at risk. This poses the risk that people living with dementia, arguably the most marginalised in any society, are likely to be the most negatively affected, with those living at the intersection of dementia and other protected characteristics being affected even more deleteriously. Policies that implement divestment from exploitative forces could be useful in enabling green, ethical and more sustainable ways forward.

Whilst the policy documents express what the growing dementia challenges are, and in many cases what is required to meet those challenges, policy overviews developed without sustained concerted political effort or accountability mechanisms and structures in place seem unlikely to be realised in a context of shrinking resources and economic retraction that seems to be a current global trend; causing the challenges posed by dementia to grow.

## Conclusion

In conclusion, it is important to be mindful that developing and implementing policy are not the complete answer to the challenges posed by dementia, and are unlikely to make meaningful incursions into the many issues facing society. There is then, a need to transform responses to those challenges in ways that cultivate a shared vision, values and practices, to secure genuinely safe and effective care for all members of society as they grow older.

## Figures and Tables

**Table I:** Themes: raising awareness, reducing stigma

The need to raise awareness about dementia, including acting to reduce the stigma around it	Dementia Services Development Centre The University of Stirling (2011), The Alzheimer Society of Ireland (2012), WHO (2012, 2015, 2017), Brodsky *et al.* (2013), HM (Her Majesty’s) Ministry of Social Affairs and Health Finland (2013), Government of Gibraltar (2015), Rubinstein *et al.* (2015), Scerri (2015), Corfield (2017), Alzheimer Europe (2016a, b, j, g, h), Norwegian Ministry of Health and Care Services (2016), Vandeurzen (2016), Hanselmann *et al.* (2017), Dementia Services Development Centre The University of Stirling (2011), The Alzheimer Society of Ireland (2012), Brodsky *et al.* (2013), Ministry of Social Affairs and Health Finland (2013), Di Fiandra (2014), World Dementia Council (2014), Rubinstein *et al.* (2015), WHO (2015, 2017), Alzheimer Europe (2016b, g, h, k), Vandeurzen (2016), Corfield (2017), Hanselmann *et al.* (2014)

**Table II:** Themes: early diagnosis through preventative, person-centred approaches, risk factors awareness raising

The need for early diagnosis, through preventative, person-centred approaches, that raise awareness of risk factors	Dementia Services Development Centre The University of Stirling (2011), HM Government of Gibraltar (2015), Alzheimer Europe (2016a, g, h), Norwegian Ministry of Health and Care Services (2016), Vandeurzen (2016), Hanselmann *et al.* (2014), WHO (2012), Ministry of Social Affairs and Health Finland (2013), World Dementia Council (2014), Rubinstein et al (2015), Alzheimer Europe (2016j, g, h), Vandeurzen (2016), Dementia Services Development Centre The University of Stirling (2011), Scottish Government (2013), Cook (2014), HM Government of Gibraltar (2015), Alzheimer Europe (2016d, j, f), Vandeurzen (2016), The Alzheimer Society of Ireland (2012), Prince *et al.* (2013), World Dementia Council (2014), HM Government of Gibraltar (2015), Rubinstein *et al.* (2015), WHO (2015, 2017), Alzheimer Europe (2016f), Norwegian Ministry of Health and Care Services (2016)

**Table III:** Themes: integrated care, fiscal investment, specialist training and education, empowerment and involvement in decision making

Avocation of integrated care models and system	Ministry of Health and Sport *et al.* (2009), Dementia Services Development Centre The University of Stirling (2011), The Alzheimer Society of Ireland (2012), WHO (2012), Scottish Government (2013), Age International (2014), Cook (2014), Di Fiandra (2014), Vandeurzen (2016), Hanselmann *et al.* (2014)
The need for increased fiscal investment	WHO (2012), Scottish Government (2013), Cook (2014), World Dementia Council (2014), Rubinstein *et al.* (2015), Scerri (2015), Alzheimer Europe (2016a, i), Vandeurzen (2016)
A requirement for continued and further research	Alzheimer’s Society (2011), Dementia Services Development Centre The University of Stirling (2011), Brodsky *et al.* (2013), Ministry of Social Affairs and Health Finland (2013), Prince *et al.* (2013), Scottish Government (2013), World Dementia Council (2014), Rubinstein *et al.* (2015), Scerri (2015), Alzheimer Europe (2015, 2016a, e, j, i, h), Manthorpe and Iliffe (2016), Prince *et al.* (2016), Vandeurzen (2016), Corfield (2017), WHO (2017)
Specialist training and education for workforces	Alzheimer’s Society (2011), Dementia Services Development Centre The University of Stirling (2011), The Alzheimer Society of Ireland (2012), HM Government of Gibraltar (2015), Rubinstein *et al.* (2015), Alzheimer Europe (2016k, a, j, h), Vandeurzen (2016)
The increased empowerment of, and support for people living with dementia, their families and their carers, including the involvement of people with dementia, their families, and carers in decision making and policy development	Dementia Services Development Centre The University of Stirling (2011), The Alzheimer Society of Ireland (2012), WHO (2012, 2015), Brodsky *et al.* (2013), Cook (2014), Di Fiandra (2014), HM Government of Gibraltar (2015), Alzheimer Europe (2016b, d, j, h), WHO (2012), World Dementia Council (2014), Alzheimer Europe (2016k, d), Norwegian Ministry of Health and Care Services (2016)

**Table IV:** Themes: care close to home

Advocating for care and support that is close to home	Ministry of Health, Welfare and Sport *et al.* (2009), HM Government of Gibraltar (2015), Alzheimer Europe (2016a, e, j)

## Notes

1. Austria, Bulgaria, Cyprus, Czech Republic, Denmark, France, Germany, Greece, Luxembourg, Poland, Portugal, Slovenia, Spain.

2. Belgium, Finland, Gibraltar, Ireland, Israel, Italy, Malta, Netherlands, Norway, Switzerland, UK England, Northern Ireland UK, Scotland UK, Wales UK.

3. Alzheimer Europe acts as an overarching organisation for Alzheimer associations across European countries. The number of associations and countries involved is growing. It holds useful information about European dementia policies.

4. Up to date information on associations and countries involved with Alzheimer’s Europe and European policies can be found at www.alzheimer-europe.org/
